# Throat Diseases—Folliculitis

**Published:** 1852-04

**Authors:** Ira Warren


*Throat Diseases—Folliculitis.* By Ira Warren, M. D. —This disease made its appearance in this country, so far as is known, in 1830, and the attention of the profession was first drawn to it as a *distinct disease* in 1832. Some have supposed its origin to have had a hidden connection with the epidemic influenza, which spread over the civilized world in 1830; but this is only conjecture. In its early developments it attracted notice chiefly by its visitations upon the throats of the clergy. Hence its popular name of *clergyman's sore throat.* It was soon found, however, to attack all classes of persons, whether engaged in any calling requiring a public exercise of the voice or otherwise. It was more noticed by public speakers and singers, by reason of the greater trouble it gave them.

The disease consists simply in a chronic inflammation of the mucous follicles or glands connected with the mucous membrane which lines the pharynx, larynx, trachea, &c. The office of these little glands is to secrete a fluid to lubricate the air passages. When inflamed, it spreads an acrid, irritating fluid over the surrounding parts, and excites an inflammation in them. This, if not arrested, ends in ulceration; the expectoration becomes puriform and undistinguishable from that of consumption, and the patient dies with all the symptoms of phthisis. Indeed before its nature was understood by the profession, it was thought the most fatal form of consumption, because it could be affected only to a very small degree, if at all, by medicines taken into the general system.

When disease lays hold of those follicles in the larynx which supply a fluid for lubricating the vocal cords, and the secretion conducted to those instruments of speech is acrid and irritating, the voice becomes hoarse; and when at length the ulceration reaches the vocal ligaments themselves, the voice suffers a gradual and finally a total extinction. I have treated a large number suffering entire loss of voice, and am happy to say it has been restored in every instance.

The approach of this disease is often so gradual as hardly to attract notice—sometimes for months or even years giving no other evidence of its presence than the annoyance of something in the throat to be swallowed or hawked up, an increased secretion of mucus, and a sense of uneasiness and loss of power in the throat after public speaking, singing, or reading aloud. At length, upon the taking of a cold, the prevalence of an epidemic influenza, or of an unexplained tendency of disease to the air passages and lungs, the throat of the patient suddenly becomes sore, its secretions increased and more viscid, the voice grows hoarse, the difficulty of speaking is aggravated, and what was only an annoyance becomes an affliction and a source of alarm and danger. The disorder clearly belongs to the family of consumption and needs early attention.

It is amusing to reflect upon the theories which writers were in the habit of constructing, a few years since, to account for the throat affection among the clergy. It was attributed by some to speaking too often, by others to speaking too loud. One class of writers thought it arose from high, stiff neck stocks; another, from a strain of voice on the Sabbath to which it was not accustomed on other days.

The cause of the disease lies deeper than any of these trifling things. So far as ministers are concerned, it may be expressed in two words—labor, anxiety.

The clerical order are placed just where they feel the force of the high pressure movements of the age. They are the only class of recognized instructors of adult men, and are obliged to make great exertions to meet the wants of their position. The trying circumstances in which they are often placed, too, in these exciting times, by questions which arise and threaten to rupture and destroy their parishes, weigh heavily on their spirits and greatly depress the vital powers. And when we add to this the fickle state of the public mind, and the shifting, fugitive character of a clergyman's dwelling place, and the consequent liability to poverty and want to which himself and family are exposed, we have a list of depressing causes powerfully predisposing to any form of disease which may prevail. As we have said, however, it is not the clergy only, but all classes of people who are afflicted with this dangerous malady.

The long and rather awkward name which Dr. Green has given to this disease is, "follicular disease of the pharyngolaryngeal membrane." I call it folliculitis, or, as this term does not describe its seat, follicular laryngitis, or follicular pharyngitis, according to its position.

Through a general lack of acquaintance with this disease, it has been often confounded with bronchitis. But bronchitis is an inflammation of the mucous membrane which lines the bronchial tubes, and of course has no existence except *below* the bifurcation of the trachea. In strictness it is not a throat disease at all.

Folliculitis is also often mistaken for laryngitis. But this latter disease is an inflammation spread over the mucous membrane of the laryngeal cavity. Bronchitis and laryngitis affect *mucous membranes;* folliculitis, the *follicles* of these membranes. Each is a separate disease, and they are easily distinguished by one who understands them. They are often complicated and unite in one subject.

There is yet another form of these chronic diseases, with which many are afflicted. Inflammation sometimes begins behind and a little above the velum palati, in the posterior nares, or back passages to the nose. Thus seated, it generally passes under the name of *catarrh in the head.* It often creates a perpetual *desire to swallow,* and gives the feeling, as patients express it, "as if something were sticking in the upper part of the throat." When the inflammation is of long standing, and ulceration has taken place, puriform matter is secreted, and drops down into the throat, much to the annoyance and discomfort of the patient. Many times the sufferer can only breathe with the mouth open. Upon rising in the morning, a great effort is generally required to clear the head, and the extreme upper part of the throat. Even distressing retching and vomiting are sometimes induced by the effort to clear the back nasal passages. There is occasionally a feeling of great pressure and tightness across the upper part of the nose; and the base of the brain sometimes suffers in such a way as to induce headache, vertigo and confusion. The smell is frequently destroyed, and sometimes the taste.

If the inflammation be in the pharynx or larynx there is a similar sensation of something in the throat, but the desire is not so much to swallow it as to hawk it up.

Besides these chronic forms of disease, there are a number of acute inflammations which attack the air passages, and run a rapid and very dangerous course. Croup is well known as one of them. There is another, which attacks the mucous membrane of the larynx and epiglottis, which reaches also the submucous cellular tissues of these organs, and which often proves fatal in a few hours. The effusion of serum into the epiglottis, in consequence of a high state of inflammation of that cartilage, causes it to stand upright, so that it cannot cover and protect the opening to the larynx; and the lips of the glottis, distended by the same cause, approach each other, thus closing up gradually the passage to the windpipe, and threatening immediate suffocation. It was this disease of which Washington died, as we learn from the clear account of the *symptoms* given by his medical attendants, though they mistook the disorder for another, the profession not being then acquainted with it.

*Treatment of Throat Diseases.*—Fifteen years ago, these disorders were thought to be incurable; and by all the appliances of medical art then known, they were so. But time has brought a successful method of treatment, as well as a clearer knowledge of their nature. The honor of first employing such treatment in this country belongs to Dr. Horace Green, professor of the theory and practice of medicine in the New York medical college. It had been previously used by Drs. Trousseau and Belloc, of Paris; but this detracts nothing from Dr. Green's just honors, as he had no knowledge of their discovery—for such it was—until after he had done the same thing on this continent.

This treatment, as is generally known to the profession, consists in topical medication, or the applying of the remedy directly to the diseased part. The medicinal agent more extensively used than any other is a strong solution of nitrate of silver. This substance is not, however, adapted to every case, other articles succeeding better in some few instances. Modern chemistry has given us a variety of articles, from which the skilful physician may select a substitute, should the nitrate of silver fail. This article has, however, proved itself nearly a *specific* for inflammation of mucous membranes, acute or chronic not connected with a scrofulous or other taint of the system ; and where such taints exist it will generally succeed, if proper constitutional remedies are used.

*Instruments.*—The instrument employed by most physicians is a piece of whale bone, bent at one end, to which is attached a small round piece of sponge. I formerly used this instrument myself, and am happy to know, that notwithstanding its defects, it was generally successful. Yet where the larynx has been highly inflamed, with a swollen and ulcerated condition of the epiglottis and lips of the glottis, I have found the singular powers of the argent. nitratis put at defiance by an irritation evidently produced by the sponge of the probang. Upon its introduction in such cases the parts contract upon and cling to it, and suffer aggravated irritation, almost laceration, upon its withdrawal, however carefully effected.

A case of this sort occurred to me in the person of a gentleman of great moral and intellectual worth, a teacher of a classical school, to whom I was called in Plymouth county, in August, 1849. He was at the point of death from starvation, not having been able to swallow anything, not even water, for a number of days. The epiglottis and lips of the glottis were much swollen and deeply ulcerated, and the whole pharyngo-laryngeal membrane involved in a high state of inflammation. The first two applications of the nitro-argentine solution, made to the isthmus of the fauces and pharynx on Saturday evening and Sunday, so far relieved him, that on Monday morning he drank, with a sense of unspeakable satisfaction, a tumbler of cold water. Before I could see him on Wednesday evening, however, he was again sinking, the full activity of the inflammation having returned ; and every subsequent attempt to introduce the sponge and to carry it down to the seat of the disease caused such irritation as to exhaust the patient. He sank and died, leaving a void in his neighborhood which it will be hard to fill. I feel confident that with the instrument I am about to introduce to the notice of the reader I could have reached the seat of the disease with so little disturbance of the parts as to have saved his life.

Such defects in the probang led me to contrive an instrument, which I call a *Laryngeal Shower Syringe.* It is in the form of a syringe, the barrel and piston of which are of glass. To this is attached a small tube, made of silver or gold, long enough to reach and enter the throat, and bent like a probang, with a globe at the end, from a quarter to a third of an inch in diameter, pierced with very minute holes, which cover a zone around the centre one-third of an inch or more in breadth.

This silver globe I daily introduce into highly inflamed and ulcerated larynges, generally without any knowledge of its presence on the part of the patient until the contained solution is discharged. A single injection throws a *very fine* stream through each of the holes in the globe, and thus all sides of the walls of the trachea are washed at once. Moreover, the smallness and smoothness of the bulb allow of its easy and painless passage through the rima glottidis, so as to bathe the walls of the trachea as low as the bifurcation, and even of the large bronchi. Physicians will understand the advantage of this in the case of ulcers low down in the trachea. They will see its advantage, too, in the case of croup in children, into whose larynges it is not easy to introduce the sponge.

The introduction of this instrument into the larynx is easy. Upon the approach of any foreign substance the epiglottis instinctively drops down upon the entrance to the larynx, guarding it against improper intrusions. It has been found, however, that when the root of the tongue is firmly depressed, this cartilage cannot obey its instinct, but stands erect, its upper edge generally rising into view. Availing himself of this fact, the surgeon has only to depress the tongue with a spatula, bent at right angles, so that the hand holding it may drop below the chin out of the way, and as the epiglottis rises to view, slip the ball of the instrument over its upper edge, and then, with a quick yet gentle motion, carry it *downward* and *forward* between the lips of the glottis, and the entrance is made. I have often admired the heroic faithfulness of this epiglottic sentinel, who, when overborne by superior force, stands bolt upright, and compels us to enter the sacred temple of speech, *directly over his head!*

This instrument I have used with great satisfaction. A considerable number of physicians in different States have procured and are now using it.

For bathing the upper part of the throat I construct it with a *straight* tube, with holes over the outer portion of the globe, and extending to the centre. This washes instantaneously the fauces and pharynx, without throwing the solution back upon the tongue.

Inflammations in the back passages to the nose have been almost entirely inaccessible by any reliable healing agent, and consequently incurable. The probang could only reach a short distance, and caused great suffering. I have had this syringe constructed with a short bend, and the globe pierced with a few fine holes at the upper end. Carrying this globe up behind the velum palati, with a single injection I wash both passages clear through. I have had the pleasure of curing a large number of bad cases of several years standing, to the surprise and delight of the patients.

Many of these throat affections are connected with functional disturbance of the liver and stomach. In such cases the inflammation of the throat generally refuses to yield until the hepatic and gastric troubles are corrected. Indeed, in a majority of cases, the topical applications need to be accompanied, for the above as well as for other reasons, by a constitutional and alterative treatment.

One word respecting the tonsils. They are chiefly "an aggregated mass of mucous follicles," and in many follicular diseases they are found enlarged, inflamed, and sometimes indurated. In such cases they secrete a thin, unhealthy, irritating fluid, which is spread over the throat, increasing and perpetuating its disease. Much of this secretion, too, finds its way into the stomach, and thence into the circulation; and I am not sure that many cases of scrofula are not engendered by the poison thus conveyed to the blood. At all events, the throat seldom gets well in such cases until the tonsils are removed.

For the excision of these glands, I found the same lack of instruments as for making topical applications to the throat. The only one which had any claims to regard was the guillotine instrument, invented some years since by Caleb Eddy, Esq., of this city. It had, however, no facilities for drawing the tonsils forward. Generally, all that could be done with it was to *trim* the gland, which did little good, for it became again enlarged. I attached the bull dog tenaculum to it, with which I have been able to draw the tonsil from between the pillars of the fauces, and cut it through the root, so as to effectually prevent a second growth. As there were still some defects in this instrument, I have prepared an entirely original one, with which the extirpation of these glands is so easy and expeditious, and withal so little to be dreaded by the patient, as to leave, I think, little further to be desired in this line.

As bearing directly upon this subject, I will add, that about three years since Dr. Chambers of London reasoned, that if nitrate of silver have a specific influence over inflammations of mucous membranes, it would cure bronchial consumption, and perhaps other forms of that disease, if it could be got into the lungs. He accordingly made a powder of that article and lycopodium, to be breathed into the lungs. His account of it was published in the London Lancet, and has appeared in this journal.

In August 1849, I prepared the same powder; and not only in the cure of bronchial consumption, but in the treatment of the *first* and *third* stages of the tubercular form of this disease I obtain results from it which I can derive from no other article.

I also use lycopodium for preparing powders in the same way with sulph. of copper, crystals of nitrate of mercury, [sometimes useful in secondary siphilitic troubles of the throat,] iodide of potassium, &c.

For breathing powders of every kind I have constructed a neat inhaler, which consists of a glass tube and a receiver—the latter being something like a tube vial, perforated with holes around the lower end. The powder is poured into the receiver, which is placed in the larger tube, and twirled between the thumb and finger while inhaling.

In the bronchial forms of consumption the local disease is confined to the mucous membranes, and in the tubercular type the deposit *begins* upon the same tissue. Breathing medicine directly into the lungs is therefore the rational mode of attacking the local disease. The time must soon come when this form of treatment will be universally adopted. The mode of applying it will doubtless be improved, and the articles employed be multiplied. But we are on the right track, and the period may not be distant when this fearful malady, taken in proper season, will be held as curable as chronic diseases of the stomach or liver,—*Boston Med. & Surg. Journal.*

